# Electrophysiological Characterization of Monoolein-Fatty Acid Bilayers

**DOI:** 10.1021/acs.langmuir.4c03814

**Published:** 2025-01-27

**Authors:** Caroline Scott, Riley Porteus, Shoji Takeuchi, Toshihisa Osaki, Sunghee Lee

**Affiliations:** † Department of Chemistry and Biochemistry, Iona University, 715 North Avenue, New Rochelle, New York 10801, United States; ‡ Artificial Cell Membrane Systems Group, Kanagawa Institute of Industrial Science and Technology, 3-2-1 Sakado, Takatsu, Kawasaki 213-0012, Japan; § Institute of Industrial Science, The University of Tokyo, 4-6-1 Komaba, Meguro, Tokyo 153-8505, Japan; ∥ Department of Mechano-Informatics, Graduate School of Information Science and Technology, The University of Tokyo, 7-3-1 Hongo, Bunkyo, Tokyo 113-8656, Japan

## Abstract

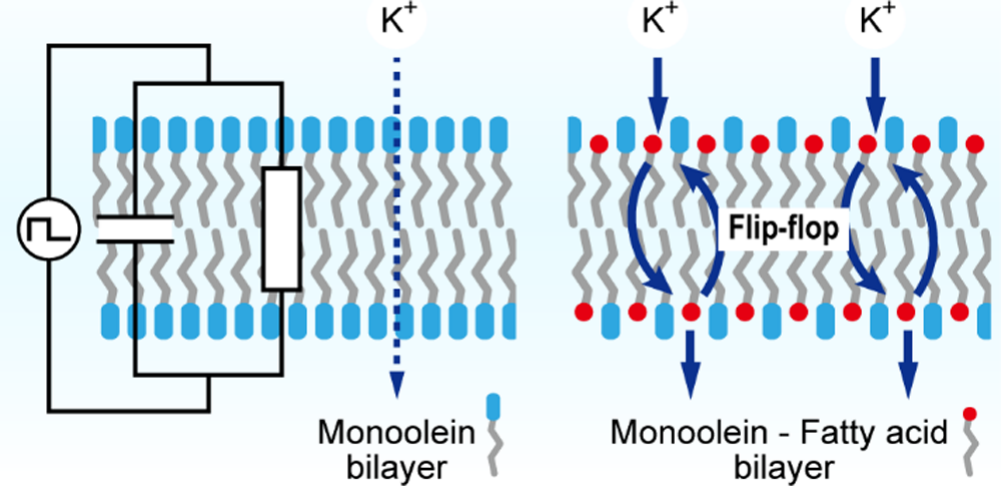

Understanding the evolution of protocells, primitive compartments that distinguish self from nonself, is crucial for exploring the origin of life. Fatty acids and monoglycerides have been proposed as key components of protocell membranes due to their ability to self-assemble into bilayers and vesicles capable of nutrient exchange. In this study, we investigate the electrophysiological properties of planar bilayers composed of monoglyceride and fatty acid mixtures, using a droplet interface bilayer system. Three fatty acids with varying hydrocarbon chain lengths—oleic acid (C18), palmitoleic acid (C16), and myristoleic acid (C14)—in combination with monoolein (C18) are examined to evaluate the influence of chain length and composition on bilayer stability, thickness, and ion permeability. The results show that pure monoolein bilayers exhibit enhanced ion permeability compared to phospholipid bilayers, which are characteristic of modern cellular membranes. Furthermore, the incorporation of fatty acids into monoolein bilayers destabilizes the membrane structure and further increases ion permeability. We consider that this increased permeability is likely driven by three molecular characteristics. First, the wedge-like shape of monoolein may disrupt bilayer packing and induce transient pore formation. Second, the rapid flip-flop of fatty acids between bilayer leaflets likely facilitates ion transport. Third, the chain-length mismatch between monoolein and myristoleic acid further destabilizes the bilayer, promoting the formation of structural defects. These findings suggest that compositional motifs in monoglyceride-fatty acid bilayers may provide an alternative ion transport mechanism, such as the flip-flop of amphiphilic molecules, in early protocell membranes before the evolution of protein-based transporters.

## 1 Introduction

The evolution of biotic systems from nonliving precursors on Earth—the origin of life—is postulated to depend critically on the development of compartments that distinguish self-from nonself.^1^ An intriguing model for such compartments is the enclosed protocell, composed of materials analogous to phospholipids that constitute evolved cellular life^2^ and likely present in the crust of prebiotic Earth. This protocell model necessitates the uptake of nutrients and the expulsion of wastes through its membrane, in addition to their growth and division. The most extensively studied model for these protocells involves aqueous-dispersed vesicles formed via the self-assembly of amphiphiles.^2^ In the epoch preceding protein evolution, it has been proposed that the transport of essential components in and out of protocells could be accommodated by passive diffusion of solutes across the membrane.^3,4^ Beyond merely transporting solutes, the membrane’s permeability must be regulated to maintain concentration gradients, thus enabling the development of thermodynamically unfavorable states.^5^ Long-chain alkyl carboxylic acids are promising candidates for such membrane components.^6^ Previous studies have demonstrated that fatty acids (FA) facilitate the self-assembly of bilayer structures and the formation of vesicles within a narrow pH range, which can grow, divide, and acquire nutrients without protein intermediacy.^7^ Furthermore, their simplicity and resemblance to modern phospholipids suggest FA vesicles as potential prebiotic compartments.^8^ Recent research has focused on mixtures of these FAs with alcohols, amines, or other moieties that enhance the stability of their vesicles.^9,10^ The incorporation of monoglycerides (MG, monoacyl fatty esters of glycerol) is known to confer stability against cations and enhances the probability of vesicle formation,^11,12^ and MG may have plausibly been present in ancient environments.^13,14^ Consequently, MG would exert a supramolecular selection pressure on the composition of protocellular bilayers. However, the electrophysiological properties, including the permeability, of mixed FA and MG bilayers have not yet been elucidated.

In this study, we investigate the effects of the composition of mixed fatty acid (FA)-monoglyceride (MG) bilayers on their electrical properties. We employ a droplet interface bilayer (DIB) system to characterize the electrical properties of FA-MG mixed bilayers. The DIB, a planar bilayer formed between a pair of water-in-oil droplets, provides a unilamellar membrane at a well-defined, isolated level,^15,16^ and serves as a versatile scaffold for measuring the electrophysiological properties of ion channel proteins^17,18^ or leaky bilayers.^19^ Here, we aim to determine the influence of FA chain length and its content on the electrical properties of MG-FA mixed bilayers. By progressively increasing the FA ratio in the bilayer, we anticipate observing changes in electrical properties that would lead to the regulation of ionic permeability suitable for a protocell model. These changes may be attributed to modulation in the packing of the bilayer, potentially revealing an intricate interplay among the bilayer components.

## 2 Experimental Section

### 2.1 Materials

**Figure 1:**
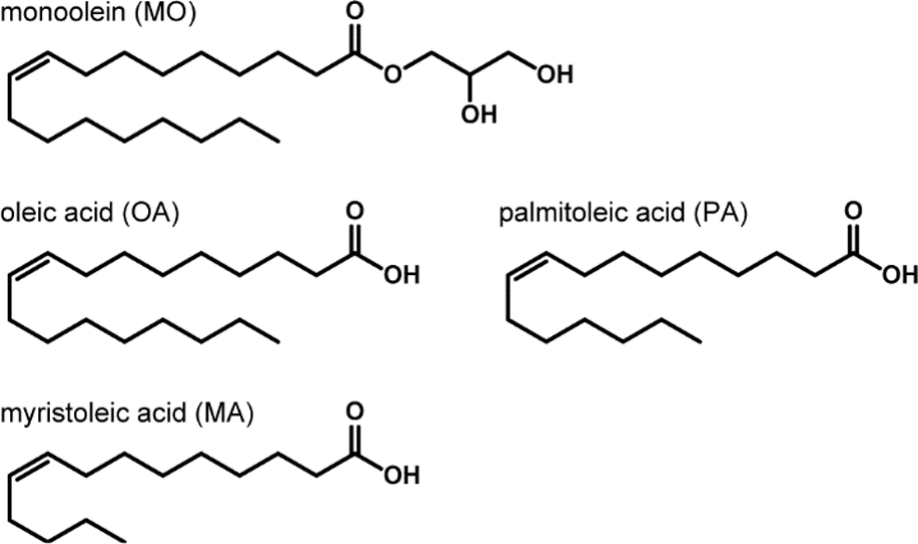
Chemical structures of MO, OA, PA, and MA.

Monoolein (MO), oleic acid (OA), palmitoleic acid (PA), and myristoleic acid (MA) were provided by Nu-Chek Prep (MN, USA) (Figure 1). The other reagents such as *n*-hexadecane, KCl, and KOH were purchased from Sigma-Aldrich (MO, USA). All aqueous solutions were prepared using ultrapure water (Milli-Q, Merck, Germany). All reagents were used without further purification. Materials for the microchip used in this study were acrylic plates (Acrylite, Mitsubishi Chemical, Japan), silver rods (Nilaco, Japan), and Ag/AgCl paste (BAS, Japan).

### 2.2 Fabrication of a Double-Well Chip

**Figure 2:**
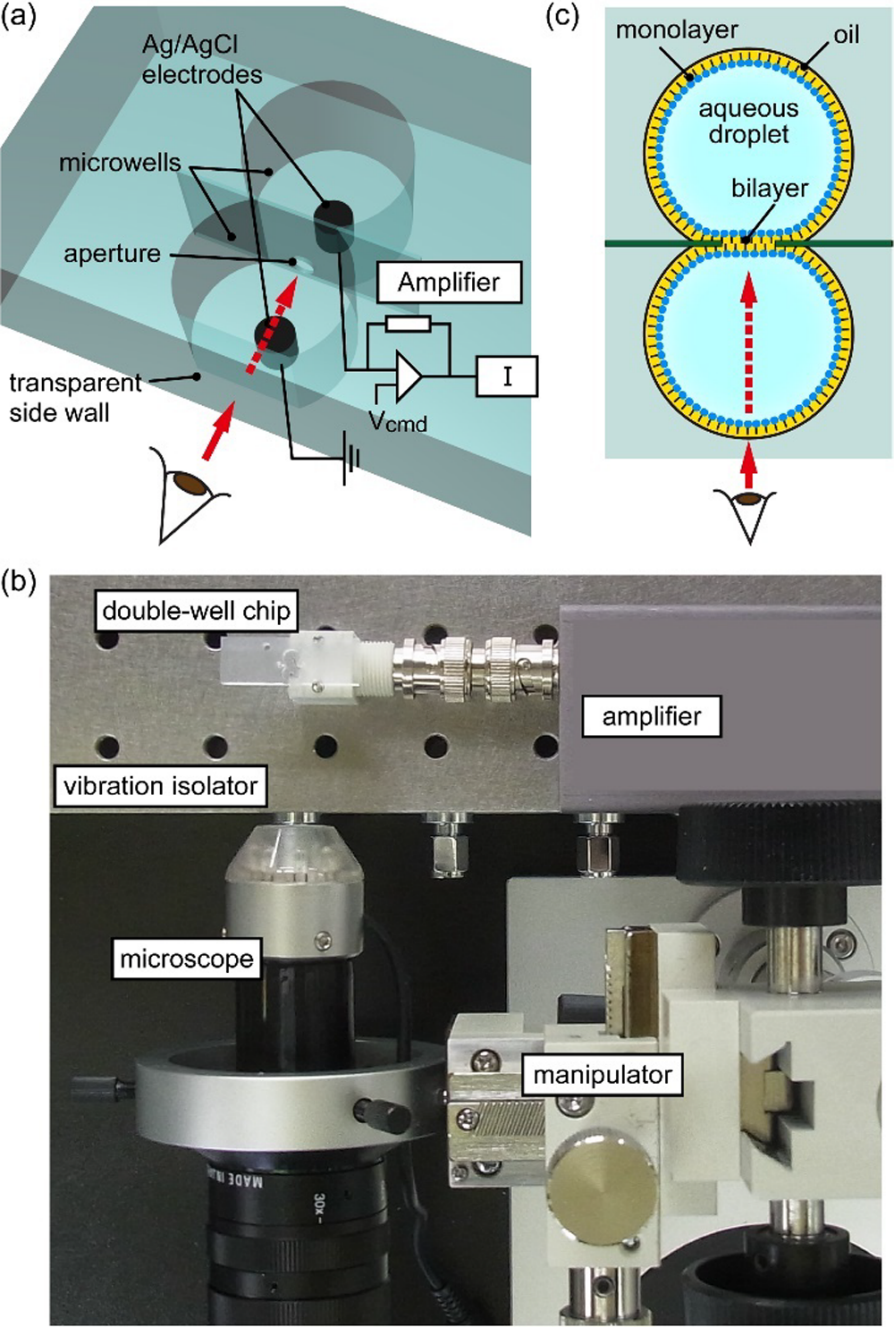
(a) Schematic diagram of the double-well microchip. (b) Overview of the experimental setup. (c) Schematic illustration of bilayer formation in the double-well chip.

A double-well microchip for membrane characterization was composed of four parts (Figure 2a)^18,20^ : an acrylic base part, a perforated separator, a pair of electrodes, and a connector. The base part was fabricated with a 4 mm-thick acrylic plate by using a computer-aided manufacturing machine (MM-100, Modia Systems, Japan). The base part consists of a pair of microwells with through-holes at the bottom. A perforated separator with a thickness of 75 μm was inserted and glued in the space between the two microwells. The aperture diameter of the separator was set to 600 μm, except for use in bilayer formation using pure FA, for which 400 μm was used. The wall outside the microwell was polished for microscopic observation of the bilayer forming at the aperture. A pair of silver rods with 1 mm diameter was embedded at the through-holes of the wells, and then assembled with a connector. Ag/AgCl paste was applied to the surface of the silver rod for electrochemical measurements. The chip was thoroughly rinsed with *n*-hexane and ultrapure water and desiccated prior to use.

### 2.3 Characterization of Membrane Resistance and Capacitance

Overview of the experimental setup is shown in Figure 2b. The double-well chip was connected to a patch-clamp amplifier (Pico2, Tecella, CA, USA). A microscope was horizontally set to observe the membrane formation at the aperture through the side wall. The bilayer region was imaged by transmitted light viewed with a digital microscope at a magnification of ×30–100 (YDZ-3F, Yashima Optical, Japan). An LED light was placed opposite the microscope. The bilayer area was evaluated from the transmitted image using ImageJ software (NIH, MD, USA). The Plateau-Gibbs border between a bilayer and an annulus was clarified by processing the image with the CLHAE algorithm (local contrast enhancement) built into ImageJ software. The clarified border was approximated with a circle and the circular area was calculated. An aluminum-foil cup was covered over the chip for a Faraday cage (not shown in Figure 2b). The amplifier and the Faraday cage were grounded to suppress the electromagnetic noise.

A desired composition of a MO and FA mixture was prepared as follows. First, MO powder and FA in liquid were respectively measured in a glass vial at a given molar ratio. Then, *n*-hexadecane was infused in the vial and the total concentration of MO and FA was adjusted at 5 mg/mL. The solution was thoroughly mixed using a vortex mixer. Prior to the experiments, 0.01 M KCl solution was freshly prepared and adjusted to pH 8.5 with 1 M KOH.

A planar bilayer membrane was formed by sequentially injecting a MO and FA mixture dispersed in *n*-hexadecane (4 μL) and the KCl solution (20 μL) in the microwells (Figure 2c). By the injections, a water-in-oil (W/O) droplet is formed in the microwell, and a monolayer with MO and FA molecules spontaneously forms at the water–oil interface. At the aperture on the separator, the two monolayers contacted each other and developed a bilayer.^21^

Membrane capacitance was obtained from the current response to a square-wave voltage stimulus. A typical response shows a peak current (*I*_max_), followed by charging the membrane capacitance (*C*_m_) with a relaxation time constant (τ), and settling to a steady-state current based on the membrane resistance (*I*_s_). The membrane capacitance is evaluated from the integration of the single-exponential current response over the time period (*t*_p_) sufficiently larger than τ.^22,23^
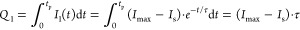
1
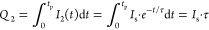
2
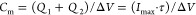
3where *Q*_1_ is the charge under the current response curve, while *Q*_2_ is added to correct the settling time of the voltage step. Here, the stimulus amplitude (Δ*V*) was set at 5 mV and the width (*t*_p_) was 100 ms. Note that τ could be assumed to be less than 1 ms based on the product of the solution resistance (<1 MΩ) and the bilayer capacitance (<1 nF). Based on the equations, *C*_m_ was automatically estimated by the software (Tecella, CA, USA). Since the membrane capacitance is the sum of the capacitances attributed to the bilayer and the annulus, the bilayer capacitance (*C*_B_) is calculated as follows.^24^
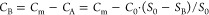
4
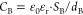
5where *C*_A_, the annulus capacitance, *C*_0_, the capacitance before a bilayer formed, *S*_B_, the bilayer area, *S*_0_, the area of the aperture, ε_0_, the vacuum permittivity, ε_r_, the dielectric constant of the bilayer, and *d*_B_, the bilayer thickness. *S*_B_ was evaluated from the microscopic image of the bilayer obtained above. ε_r_ was set to 2.1 and *d*_B_ was estimated with eq 5.^25^

Membrane resistance was estimated from the current vs voltage plot. Representative ionic current vs applied voltage plots are shown in Figure S3. The ionic current in steady state was measured under the application of DC voltages (0, ±10, ±20, and ±30 mV). The bilayer resistance was determined from the product of the bilayer area and the inverse of the slope evaluated by linear fitting of the plot.

## 3 Results and Discussion

### 3.1 Bilayer Formation with MO-FA Mixtures

We confirmed the process of bilayer formation with various monoolein (MO) and fatty acid (FA) compositions by microscopic observation of the Plateau-Gibbs border between a bilayer and an annulus.^26^ We chose MO as a representative of monoglycerides because its carbon chain length of 18 is the same as that of phospholipids commonly used for bilayer formation. As a counterpart to MO, we selected three FAs of oleic acid (OA), palmitoleic acid (PA), and myristoleic acid (MA) with chain lengths of 18, 16, and 14 (Figure 1). Five molar ratios of 100–0, 50–50, 30–70, 15–85, and 0–100 MO-FA were examined.

**Figure 3:**
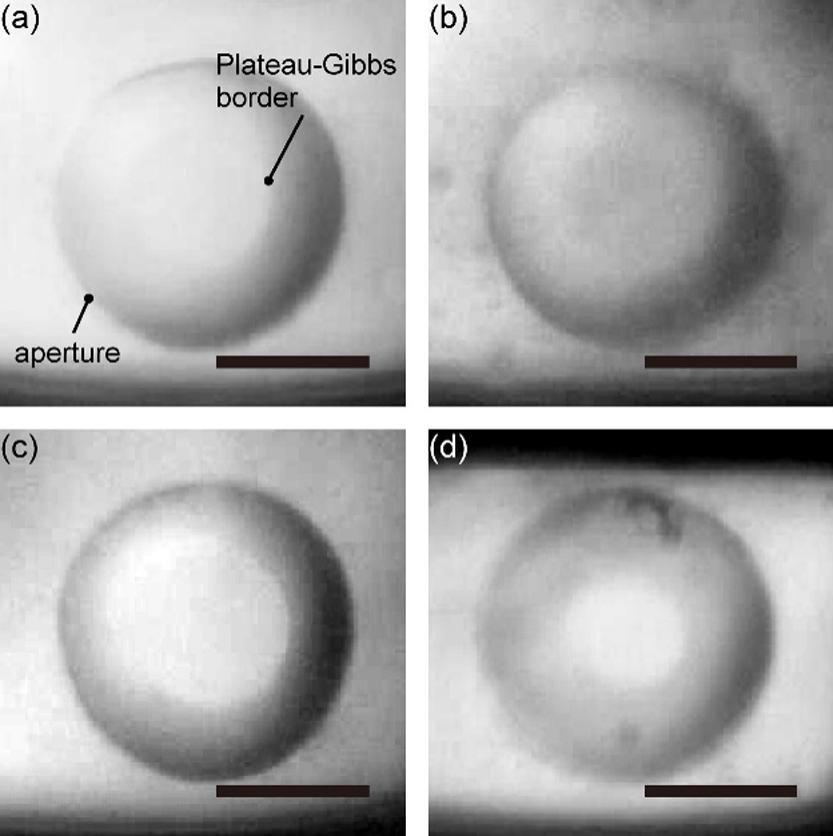
Microscopic images of the bilayers formed with the MO-FA mixtures. (a) 100% MO, (b) 15–85 MO-OA, (c) 15–85 MO-PA, and (d) 15–85 MO-MA. Brightness and contrast were adjusted for visibility. Scale bar: 300 μm.

Typical microscopic images of the MO bilayer and MO-FA bilayers are shown in Figure 3. For most of the compositions, a Plateau-Gibbs border was observed after the attempt of bilayer formation, indicating that these MO and MO-FA mixtures were able to form a planar bilayer membrane with *n*-hexadecane solvent. We previously confirmed that pure monoglycerides form stable and long-lived bilayers.^27,28^ We additionally verified the bilayer formation by capacitance measurements below. It should be noted that the bilayer formation process often failed with increasing the FA ratio, and pure MA rarely formed a bilayer with the microchip under the conditions in this study (Table S1–1 provides success rate of bilayer formation). For the formation of pure OA and PA bilayers, we used a 400 μm diameter aperture to stabilize the bilayers,^21^ while 600 μm diameter was used for all other compositions. The success rates of pure OA and PA bilayers were consistently lower than the pure MO bilayer, as observed through our empirical experience (Table S1–1). Moreover, the formed bilayer became unstable with increasing the FA ratio, and the pure OA and PA bilayers were only maintained for the minimum time required for capacitance and resistance measurements (approximately 3–4 min). We consider that the bilayer formation process with pure FA, i.e., the contact of two FA monolayers at the aperture, was difficult to proceed, and even after the formation of a planar bilayer, the bilayer exhibited fragility and was susceptible to rupture.

### 3.2 Capacitance and Thickness of MO-FA Bilayers

**Figure 4:**
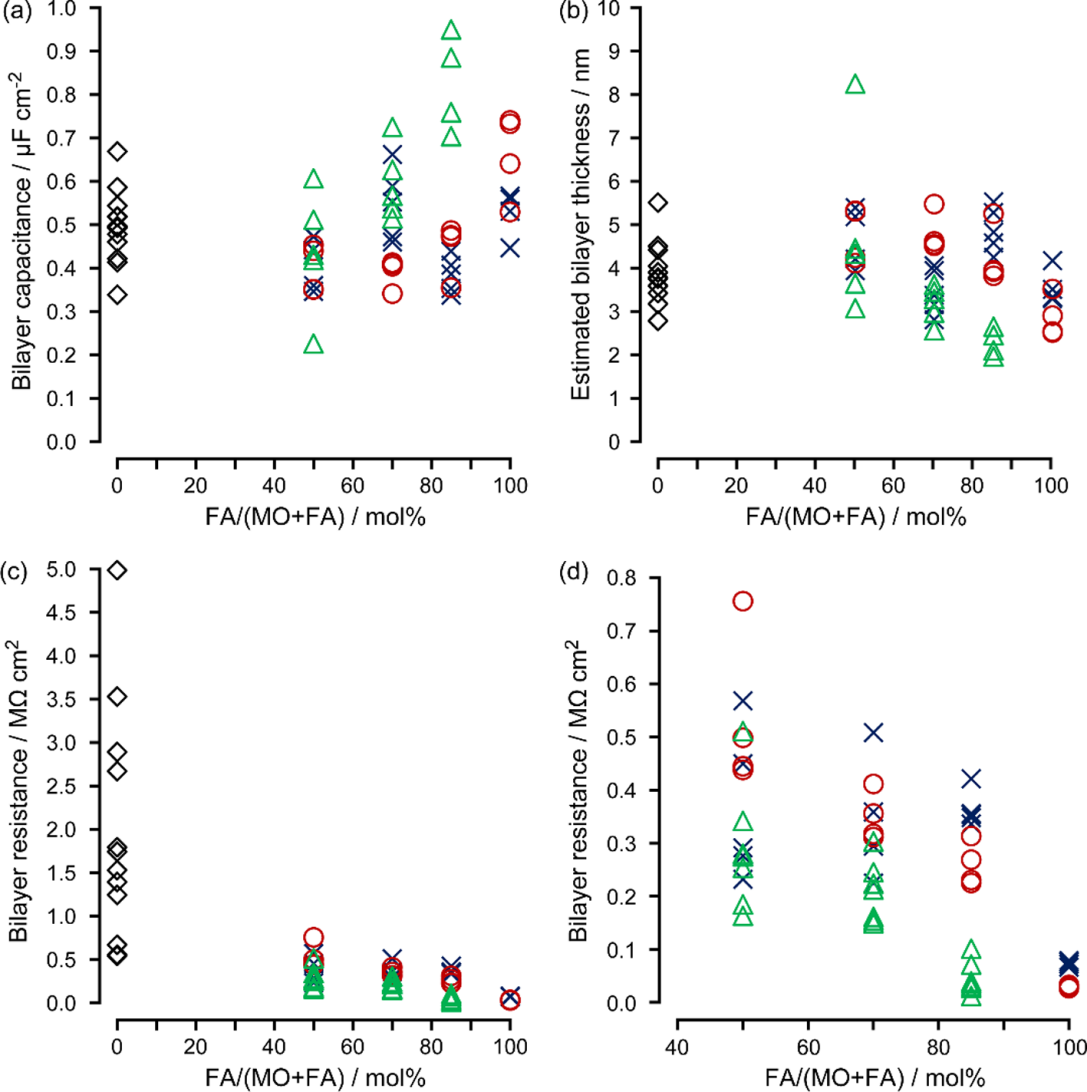
(a) Bilayer capacitance, (b) estimated bilayer thickness, (c) bilayer resistance, and (d) an enlarged view of bilayer resistance depending on the molar ratios of FAs. 100% MO (black diamond); MO-OA (blue cross); MO-PA (red circle); MO-MA (green triangle). Each point represents the data obtained from individual bilayers (*N* ≥ 4 for each MO-FA composition).

To verify the bilayer formation with the varied MO-FA compositions, we measured the membrane capacitances and calculated the bilayer thicknesses. As described in Section 2.3, the capacitance was obtained from the response to the application of a square pulse to the membrane. The thickness of the bilayer was estimated from the capacitance and the area within the Plateau-Gibbs border evaluated from the microscopic image. Figure 4a,b represent the capacitance and estimated thickness of the formed bilayer as a function of the MO-FA ratio. The corresponding capacitance and thickness values are provided in Table S1–2. The capacitance range between 0.5 and 1.0 μF/cm^2^ was in the same order of magnitude as that of a phospholipid bilayer previously reported,^24^ and agreed with those of the monoglyceride bilayers.^29^ The thickness of pure MO bilayer was also compatible to that of a phospholipid bilayer with the chain length of 18 (Figure 4b).^30^ Along with the observation of the Plateau-Gibbs border, we consider that these MO-FA mixtures were capable of forming a planar bilayer membrane. Regardless of the molar ratio of OA to MO, the capacitances and thicknesses were not significantly different; capacitances ranged between 0.38 ± 0.04 to 0.55 ± 0.08 μF/cm^2^ (see Table S1–2 for data and Figure S1 for statistical evaluation). On the other hand, the MO-MA bilayer exhibited a larger capacitance (0.44 ± 0.14 to 0.82 ± 0.11 μF/cm^2^) and a thinner bilayer with an increasing ratio of MA (Table S1–2 and Figure S1).

### 3.3 Resistance of MO-FA Bilayers

We evaluated the resistance of the MO-FA bilayers to characterize the ionic permeability through these bilayers (see Table S1–2 for data and Figure S2 for statistical evaluation). The bilayer resistance was obtained from the slope of the current vs voltage plots (Figure S3). Figure 4c shows the resistances as a function of the MO-FA ratio. The bilayer resistance of approximately 2 MΩ cm^2^ with pure MO was in good agreement with the planar bilayer data previously reported.^19,29^ This resistance was found to be 1 order of magnitude lower than that of a planar phospholipid bilayer (the latter being, typically, >10 MΩ cm^2^).^31^ Moreover, the bilayer resistance decreased with increasing the FA ratio, and the resistances of pure OA and PA bilayers were less than 0.1 MΩ cm^2^ (Table S1–2). These results exhibited the relatively large ionic permeability of the MO-FA bilayers compared to phospholipid bilayers. Note that the MO-MA bilayer with a thinner thickness showed a lower resistance than the MO-OA and MO-PA bilayers. According to the resistance value, a few billion ions per second pass through the pure MO planar bilayer of 300 to 400 μm in diameter. This value of conductance for pure MO bilayer can be compared to the amount which would have been engendered had an ion channel protein been responsible for the observed current. Converting to a typical ion channel protein with a conductance of 20 pS, it is estimated that there would be one ion channel per 200 μm^2^ for the pure MO bilayer, which is a significantly small number of ion channel proteins compared to a living cell of 10 μm in size (approximately 300 μm^2^ surface area).

The MO bilayer exhibited significantly higher permeability (1 order of magnitude lower resistance, Figure 4c and Table S1–2) compared to a phospholipid bilayer which is characteristic of modern cellular membrane. This permeability difference cannot be attributed to differences in hydrocarbon chain length or bilayer thickness, as shown in Figure 4b. We consider that the difference lies in the packing state of the bilayer between MO and phospholipids. As illustrated in Figure 1, the monoolein molecule contains a *cis* double bond at the ninth position, which induces steric hindrance in the hydrocarbon chain. Considering the size of its headgroup and its single alkyl chain, the molecule exhibits a wedge-shaped (inverted cone) geometry.^32^ This shape favors the formation of a bilayer with negative curvature during self-assembly. Contrarily, phospholipids, such as a phosphocholine, possess a cylindrical shape due to the balance between their headgroup and dual hydrocarbon tails, which is conducive to the formation of a planar bilayer with near-zero curvature.^33^ The wedge-like geometry of MO molecule likely disrupts the tight packing typically observed during bilayer assembly. This disruption, coupled with the presence of a *cis* double bond in MO’s structure, could inhibit cohesive interactions between the hydrocarbon chains.^34^ Consequently, this may facilitate the permeation of ions and water molecules through the bilayer.

In our study, the investigated FA molecules possess the same monounsaturation but differ in hydrocarbon chain length. The presence of a ‘kink’ in the FA’s structure, due to its *cis* double bond, increases the splay of its hydrocarbon chains. This could further disturb the intermolecular order and create packing defects in the MO-FA bilayer structure. The addition of OA increased the ionic permeability (decreased the resistance) of the MO-OA bilayers (Figure 4c), suggesting that the molecular characteristics of OA play a crucial role in determining permeability, even though the bilayer thickness remained relatively unchanged (Figure 4b). We consider that the increased permeability at no significant change in thickness can be attributed to the respective natures of capacitance and resistance measurements: capacitance reflects an averaged structural arrangement of the entire bilayer, whereas resistance data provides a snapshot of local ionic permeation events. The flip-flop of FA molecules may contribute to ionic transport across the bilayer. Previous studies have reported that the flip-flop of ionized OA occurs with a time constant of a few minutes;^35,36^ pertinently, it was demonstrated that fatty acids facilitate the flux of potassium across the bilayer by association of the monovalent cation to the carboxylate anion of the fatty acid followed by transmembrane flip-flop. This time constant is significantly faster than that observed in phospholipid bilayers.^37^ We performed a rough estimate of the number of ionized OA molecules flipping across a MO-OA bilayer per second and compared the result with the number of ions permeating the bilayer based on the resistance result (See Suppl. Text S1 for details). Assuming a bilayer diameter of 250 μm, approximately 10^8^ ionized OA molecules flip from one leaflet to the other every second. This number is on the same order of magnitude as the ion permeation estimated from bilayer resistance, suggesting that ionized OA may directly facilitate ion transport during flip-flop or that a transient pore may be formed within the bilayer as a result of the flip-flop. In addition, we observed no discontinuities in resistance or capacitance as the fatty acid ratio increased. These continuous trends in membrane properties suggest a uniform integration of fatty acids into the monoolein bilayers.

A further increase in permeability was observed for the MO-MA bilayers compared with the MO-OA and MO-PA bilayers. Correlating with the decrease in bilayer thickness, the enhanced permeability should be attributable to the shorter hydrocarbon chain length of MA. Packing defects can arise from mismatches in the hydrocarbon chain region, as demonstrated in mixed phospholipid membrane systems.^38,39^ In our system, a significant chain length mismatch exists between MO (C18) and MA (C14). To compensate for the hydrophobic “voids” in the bilayer interior caused by this mismatch, the longer hydrocarbon chains from both leaflets may tilt or bend within the bilayer matrix. Such adaptation creates packing defects in the bilayer interior, leading to increased fluidization and pore formation, which would facilitate the flip-flop of MA molecules, as evidenced by the increased ion permeability observed with higher MA concentrations. Owing to the presence of a *cis* unsaturation in the MO molecule, tilting and bending to fill these voids is a more likely scenario rather than any interdigitation (i.e., accommodating MO chains into the opposite leaflet). Were the latter to be the case, an increase in rigidity would be expected, which is not observed.

In our system, one cannot discount the involvement of the hydrocarbon solvent (hexadecane) in the process of ionic conductivity. While hexadecane, when used in reconstituting lipid bilayers, has a lesser propensity for remaining within the bilayer structure as compared with other hydrocarbons typically employed (such as decane), its presence in the monoolein bilayer is reportedly substantial, at a volume fraction of 0.08.^40^ Fortuitously, recent molecular dynamics (MD) simulation studies have investigated the impact of hexadecane solvent on reconstituted bilayer properties, showing that the presence of hexadecane oil only marginally affects surface properties, membrane order, and lateral stress, factors which could have a bearing upon pore formation and/or lipid flip-flop.^41^ Still, the rapidity with which hydrocarbon solvent can diffuse within the lipid bilayer should influence ionic conductivity, owing at least to the possibility of repair of transient pores by solvent molecules. Nevertheless, despite a residual presence of some hexadecane, we believe that our sequence of studies constitutes a fair comparison, given the fact that the same solvent is present in each run. Additionally, we observed that mixed bilayers of MO/OA and MO/PA have the same capacitance (therefore thickness), suggesting that any content of hexadecane in these respective systems is essentially the same and thus controlled. Therefore, the differences in ion permeability that we observed is meaningful even in the presence of some solvent. It will be a focus of future studies to compare different types of organic solvents and their associated changes in the properties of the artificial membranes. Moreover, it will prove even more fruitful to investigate the role of solvent molecules by constituting rigorously solvent-free bilayers, using a Montal-Mueller (“folding”) method,^42^ or more effective recent techniques.^43^

## 4 Conclusions

In this study, we clarified the electrical properties of monoolein-fatty acid bilayers using a double-well microchip system. We examined three fatty acids with varying hydrocarbon chain lengths: oleic acid (C18), palmitoleic acid (C16), and myristoleic acid (C14). The inclusion of monoolein was found to stabilize the planar bilayers formed by these fatty acids. The ion permeability of the monoolein bilayer was an order of magnitude higher than that of a phospholipid bilayer of similar chain length, likely due to the disruptive effect of wedge-like molecular shape of monoolein. Increasing the fatty acid content in the fatty acid/monoolein bilayers resulted in a further rise in ion permeability, independent of bilayer thickness, although the inclusion of the MA, which has a shorter chain length, led to a concurrent increase in permeability and a decrease in the thickness of the MO-MA bilayers. This enhanced permeability was probably attributed to the rapid flip-flop movement of fatty acid molecules between the bilayer leaflets, which facilitates ion transport. Moreover, the chain length mismatch between monoolein and the fatty acid appeared to destabilize the bilayer, contributing to an additional increase in ion permeability.

These findings suggest that such compositional variations in bilayers can modulate ion permeability in a manner consistent with models of early protocell membranes. Given that transmembrane transport in these primitive systems would rely on nonenzymatic mechanisms, such as the formation of structural defects and transient pores, our results provide potential evidence for the role of flip-flop in directly or indirectly facilitating ion transport across protocell membranes composed solely of single-chain amphiphilic molecules.

## Supporting Information Available

Theoretical estimation of fatty acid flip-flop rate within a bilayer; estimation of ion permeation from membrane resistance; averaged bilayer capacitances as a function of the molar ratios of FAs; averaged bilayer resistances as a function of the molar ratios of FAs; representative ionic currents vs applied voltage plots for MO-FA bilayers; success rate of bilayer formation; and average and standard deviation of the electrical properties of MO-FA bilayers (PDF)

## Abbreviations

FA: fatty acid
MG: monoglyceride
DIB: droplet interface bilayer
MO: monoolein
OA: oleic acid
PA: palmitoleic acid
MA: myristoleic acid
